# How do bacterial membranes resist polymyxin antibiotics?

**DOI:** 10.1038/s42003-020-0803-x

**Published:** 2020-02-17

**Authors:** Adree Khondker, Maikel C. Rheinstädter

**Affiliations:** 1 Department of Physics and Astronomy, McMaster University, Hamilton, ON Canada; 2 Faculty of Medicine, University of Toronto, Toronto, ON Canada; 3 Origins Institute, McMaster University, Hamilton, ON Canada

**Keywords:** Microbiology, Membrane biophysics

## Abstract

In our recent Communications Biology article, we reported on the biophysical mechanism of resistance for polymyxin antibiotics in bacterial membranes. The emergence of plasmid-borne colistin resistance poses a threat to our last line of defense against many pathogens. Here, we outline the current understanding of *mcr-1*-mediated polymyxin resistance, and propose future directions for membrane-targeting antibiotic research.

## Abstract (web summary)

Adree Khondker and Maikel Rheinstadter discuss how bacteria escape being killed by polymyxin antibiotics. Touching on their recent Communications Biology paper, they elaborate on the mechanism by which the bacterial membrane becomes resistant and on future directions to take in order to understand this phenomenon.

Polymyxin antibiotics, such as colistin, have important roles in both medicine and agriculture. However, the use of polymyxins for the latter endangered its use in the former. The first transmissible plasmid-bearing resistance to colistin was reported by Liu and colleagues in 2016, and isolates of *Escherichia coli* harboring this resistance have been found in livestock across southeastern Asia^1^. Expression of the resistance gene, *mcr-1*, causes modification of lipid A in the bacterial outer surface, resulting in reduced affinity for polymyxins. Worryingly, the gene can be readily passed between different bacterial strains making widespread polymyxin resistance inevitable^2^. Wang and colleagues reported on the global distribution of *mcr-1* and showed the resistance gene is currently present across five continents, as shown in Fig. 1 (ref. ^3^).

**Fig. 1:**
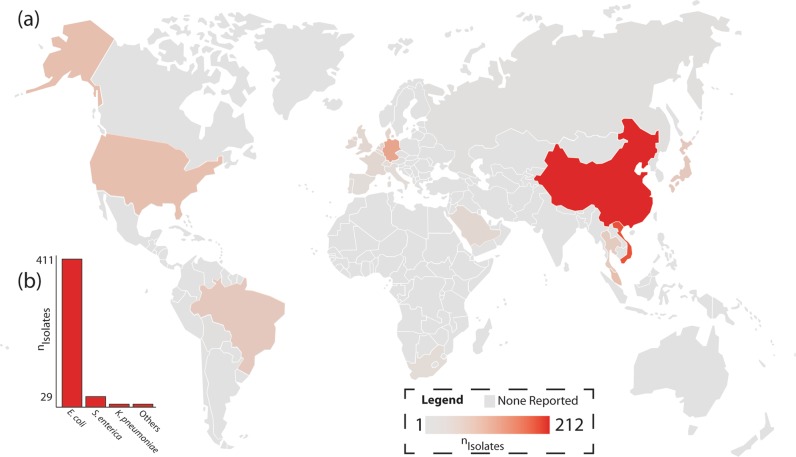
Geographic distribution of polymyxin resistance. **a** Global incidence of *mcr-1*-harboring isolates per country and **b** by bacterial strain, data used from ref. ^3^.

Polymyxin antibiotics kill bacteria by damaging their cell membranes, but now bacteria have figured out how to inhibit this process. Biophysical tools, such as diffraction and molecular dynamics computer simulations, have provided important insights into these mechanisms^4,5^. In order to prolong the lifespan of current polymyxins and develop new ones, it is critical to gain a detailed understanding of the biophysics of polymyxin–bacteria interactions.

## How do polymyxins damage bacterial membranes?

**Fig. 2:**
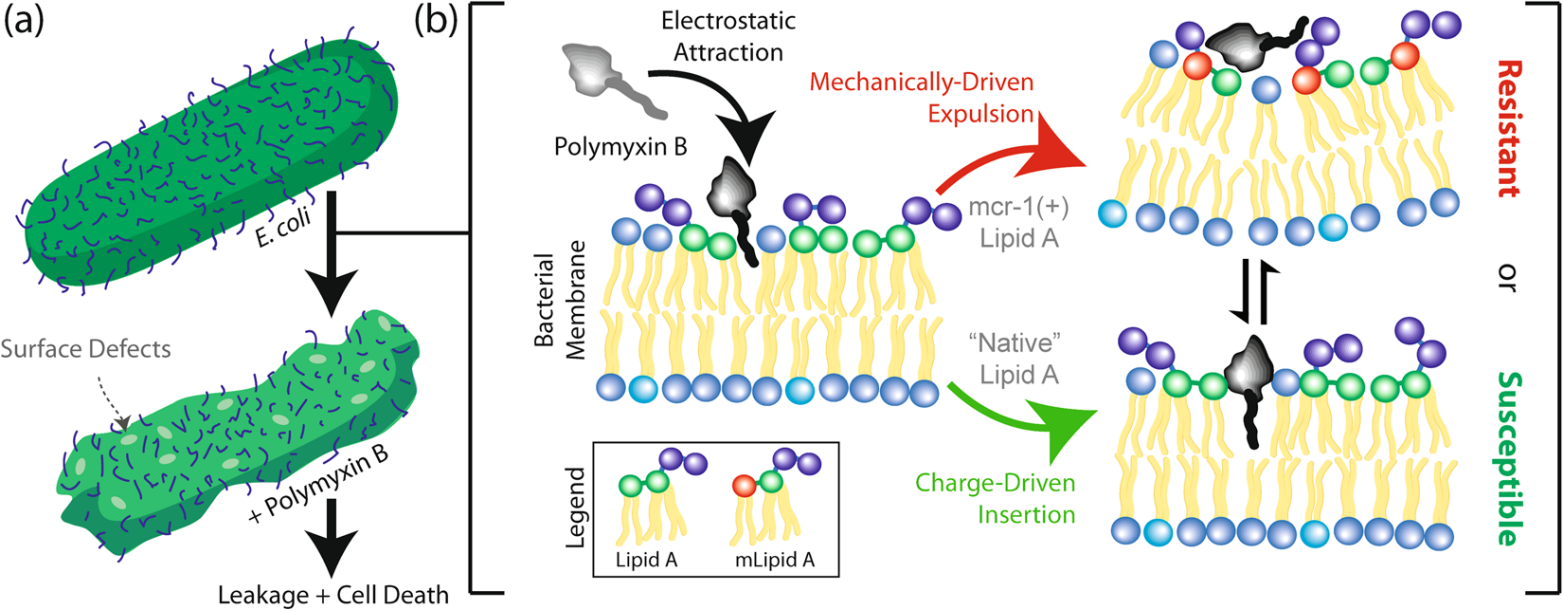
Mechanism of resistance for polymyxin antibiotics conferred by mcr-1. **a** General mode of polymyxin activity, and **b** biophysical mechanism of action for polymyxin-induced membrane damage in general membranes, as described in Khondker et al.^11^. mLipid A is modified lipid A.

The earliest high-resolution studies showed that polymyxins can kill bacteria by puncturing holes into their outer surface, and causing leakage of internal contents^6–8^. Polymyxin B and colistin are both cationic antimicrobial peptides that are attracted to the net anionic bacterial outer membrane, resulting in an electrostatic attraction between drug and target. The polymyxin creates local curvature in the membrane, while the membrane itself repels the insertion process. At a critical point, the hydrophobic tail of the polymyxin can insert into the bacterial membrane and create a lipid defect by separating lipids away from one another. When fully inserted into the membrane core, the polymyxins are highly mobile and start forming aggregates in the membrane core, leading to the increased water intake and structural instabilities. The accumulation of those defects in the membrane eventually results in the permeation of water across the bilayer, dysfunction of membrane proteins, the formation of membrane pores at high concentrations, and subsequent membrane collapse. This mechanism has been supported experimentally by our work and others^9–12^, and is pictured in Fig. 2. The higher the net negative bacterial membrane charge, the more susceptible bacteria are to the formation of these defects.

Indeed, increasing polymyxin concentration is proportional to membrane damage and bacterial cell death; however, there are inconsistencies at higher concentrations with regards to damage. Currently the pore formation models attribute these to aggregation effects, or polymyxin pores in the form of a barrel stave. At high concentrations of polymyxin, the polymyxin molecules will form aggregates on top of the bacterial surface that can create large physical defects via the carpet model of insertion^5,9,13^. One of our current goals is to sensitively measure concentration-dependent effects of polymyxin on membrane damage using a lipid-based biosensor that detects passivating currents through a membrane layer in polymyxin-enriched environments. These data will be important to determine threshold concentrations that may be necessary for bactericidal effects.

The nonspecific nature of polymyxin interactions with membranes occurs in the absence of biochemical binding to specific targets. This can also lead to unwanted side effects through the damage of renal epithelial cells, giving rise to nephrotoxicity. We have previously reported that membrane cholesterol, for instance, is crucial for the suppression of polymyxin-induced damage in kidney membrane mimics^9^. Cells in the renal papillary ducts, which do not contain stiffening membrane cholesterol, seem especially susceptible to polymyxins. Whereas cholesterol did not significantly prevent polymyxin insertion into the membrane, it prevented membrane collapse and subsequent cell death by stabilizing the membrane structure.

Combining colistin with numerous antibiotics have shown synergistic effects, and this offers a promising approach to overcome bacterial resistance. Specifically, clarithromycin in combination with polymyxin showed efficacy in mouse models to eliminate *mcr-1* infection and improve survival^14^. The two biophysical processes to explain this phenomenon likely involve either a direct drug–drug interaction between polymyxin and other antibiotics or that the damage done to the bacterial membranes increases permeability to antibiotics with intracellular targets.

## How are resistant membranes different?

The two balancing forces that determine whether polymyxins can insert into membranes and create damage are electrostatic attraction and the “elastic” resistance of the membrane against penetration. If the charge difference is larger than the repulsive forces, the polymyxin antibiotic will eventually be able to penetrate and create membrane damage. Liu and colleagues showed that in *mcr-1*-expressing bacteria, a negatively charged phosphate on each lipid A in the bacterial outer membrane is replaced by a small neutral ethanolamine moiety in highly virulent pathogens^15^. The loss of a negatively charged group in the bacterial membrane reduces the affinity of the cationic polymyxin. Moreover, addition of the ethanolamine to lipids across the bacterial surface increases the volume of the membrane core and intermolecular attraction between adjacent lipids, ultimately increasing membrane stability and resistance to mechanical compression and membrane collapse. Paracini et al. showed that polymyxin B activity is dependent on a gel to liquid crystalline phase transition in complex membrane models, and modifications to the structure of lipid A plays a determining role in the phase of the bacterial outer membrane^16^. Altogether, *mcr-1* expression was found to affect the global physical properties of bacterial membranes, making resistant bacteria less attracted to the polymyxin, and less susceptible to polymyxin insertion (Fig. 2).

Notably, polymyxin resistance can also be triggered by two-component signal transduction systems, such as PmrAB and PhoPQ, in response to environmental conditions, such as irregular local cation concentrations^17–19^. In *Acinetobacter baumannii*, polymyxin resistance can be conferred by complete loss of negatively charged lipopolysaccharide on the membrane surface^20^. The physical principles behind the resistance is similar; the loss of the charge and increase in membrane rigidity will independently or synergistically confer resistance to polymyxin antibiotics.

We are currently also focusing our attention on the similarities between polymyxins and other membrane-damaging antibiotics. The underlying mechanisms of these antibiotics are likely also concentration dependent, with a regime where defects are created, and another where pores form in the bacteria cell walls. A unified model may explain the contrasting results with regards to different mechanisms of polymyxin resistance.

## Concluding remarks

Polymyxin antibiotics have provided a critical option for clinicians in treating complex multidrug-resistant infections. With advances in biophysical imaging techniques and increasing computational power, it has become possible to observe each subsequent step from polymyxin binding to membrane damage, while measuring the physical effects on the structure of the bacterial outer membrane. With a better understanding of strain-specific resistance, novel lipopeptides based on the polymyxins may be developed, which also overcome toxicity concerns. Fortunately, we are seeing these derivatives in preclinical studies, and increased attention from the biophysical community on developing antimicrobial peptides as a whole^21,22^. Li, Nation, and Kaye have recently edited the book “Polymyxin Antibiotics: From Laboratory Bench to Bedside”, which provides a comprehensive understanding on the current state of polymyxin antibiotics^23^.

With regards to the urgency of polymyxin resistance, it is important to consider what can be done on a short term. Indeed, one must restrict polymyxin use in agriculture, or we will soon find ourselves without our last-line defense against multidrug-resistant infections.
